# Radiation-induced morphoea in the setting of previous breast cancer: A case report

**DOI:** 10.1177/2050313X261463846

**Published:** 2026-07-08

**Authors:** Sydney A. Yee, David M. Langelier, Kyla Taylor, Lynne H. Robertson

**Affiliations:** 1 Department of Medicine, Cumming School of Medicine, University of Calgary, AB, Canada; 2 Division of Clinical Neurosciences, Department of Medicine, Cumming School of Medicine, University of Calgary, AB, Canada; 3 Division of Oncology, Department of Medicine, Cumming School of Medicine, University of Calgary, AB, Canada; 4 Department of Pathology and Laboratory Medicine, Cumming School of Medicine, University of Calgary, AB, Canada; 5 Division of Dermatology, Department of Medicine, Cumming School of Medicine, University of Calgary, AB, Canada

**Keywords:** radiation-induced morphoea, morphoea, radiation therapy, breast cancer

## Abstract

Radiation-induced morphoea is an under-recognised complication of radiotherapy that can cause significant pain, functional impairment, and disfigurement. It is frequently misdiagnosed due to its non-specific presentation. We report the case of a 70-year-old female with prior right breast invasive ductal carcinoma, status post-lumpectomy, who developed painful cutaneous thickening and atrophy within the irradiated field 18 months after adjuvant radiotherapy. Malignant recurrence was excluded, and biopsy revealed dermal sclerosis with collagen thickening consistent with radiation-induced morphoea. Management included intralesional corticosteroid injections, regional nerve blocks, and physical therapy. Early recognition, biopsy, and multidisciplinary management are critical to improving patients’ quality of life.

## Introduction

Radiation-induced morphoea (RIM) is a rare fibrosing complication, commonly reported in breast cancer patients after completing radiotherapy.^1^ It is associated with substantial pain and disfigurement, greatly impacting quality of life. However, RIM is frequently misdiagnosed as cellulitis or cancer recurrence due to its non-specific presentation, ultimately delaying treatment.^2^ Here, we present a case of RIM, which highlights the importance of early recognition with biopsy and implementation of a multidisciplinary team to improve patient outcome.

## Case report

A 70-year-old female, who was treated successfully for estrogen receptor (ER)/ progesterone receptor (PR) positive, human epidermal growth factor receptor 2 (HER-2) negative breast cancer, was referred to dermatology for evaluation of rapidly progressive sclerotic changes of the right breast skin and underlying tissues.

In 2021, she was diagnosed with ER/PR-positive stage 1A invasive ductal carcinoma of the right breast. She underwent a lumpectomy and adjuvant whole breast radiotherapy with a total dose of 26 Gy over five fractions. There were no major acute side effects, although she did develop Grade II skin reactions to radiotherapy that resolved with topical steroid therapy. She was also briefly started on anastrozole, followed by letrozole, but these were discontinued due to intolerance. At the time of presentation to dermatology, her cancer was under surveillance.

Eighteen months after radiotherapy completion, she developed cutaneous thickening with violaceous to pink discolouration and underlying tissue atrophy of the right breast. This was associated with substantial pain across the right anterolateral chest extending towards the axilla and lateral chest wall, which was not in keeping with post-mastectomy pain syndrome alone. There was no evidence of lymphoedema. Mammogram and magnetic resonance imaging showed diffuse oedema and thickening of the breast skin, but no focal abnormality. Biopsies were also negative for recurrent or new malignancies.

**Figure 1.**
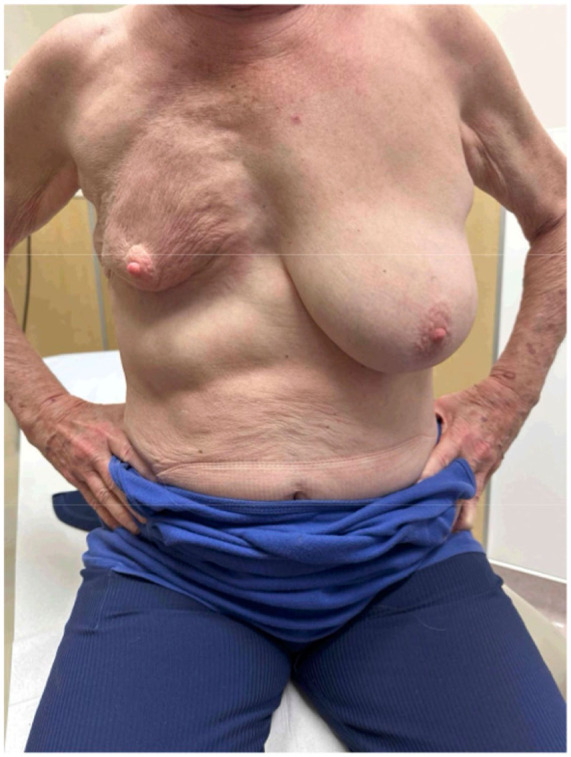
Sclerotic atrophy with associated erythematous areas of peau d’orange of the right breast tissue in a 70-year-old female after 18 months of radiotherapy. Affected areas were limited to radiotherapy sites and did not impact the left breast.

Cutaneous examination revealed a well-demarcated, sclerotic, atrophic, very firm plaque with marked atrophy of the underlying right breast tissue and multiple thickened bands throughout the right breast (Figure 1). The overlying skin demonstrated wrinkling, areas of peau d’orange, and erythema, sparing the nipple/areola. The remaining ipsilateral breast was significantly smaller in size compared to the unaffected side. Tissue changes were confined to the site of previous radiation therapy as per her radiation tattoos. There were no other areas of sclerosis, induration, or erythema on the skin.

**Figure 2.**
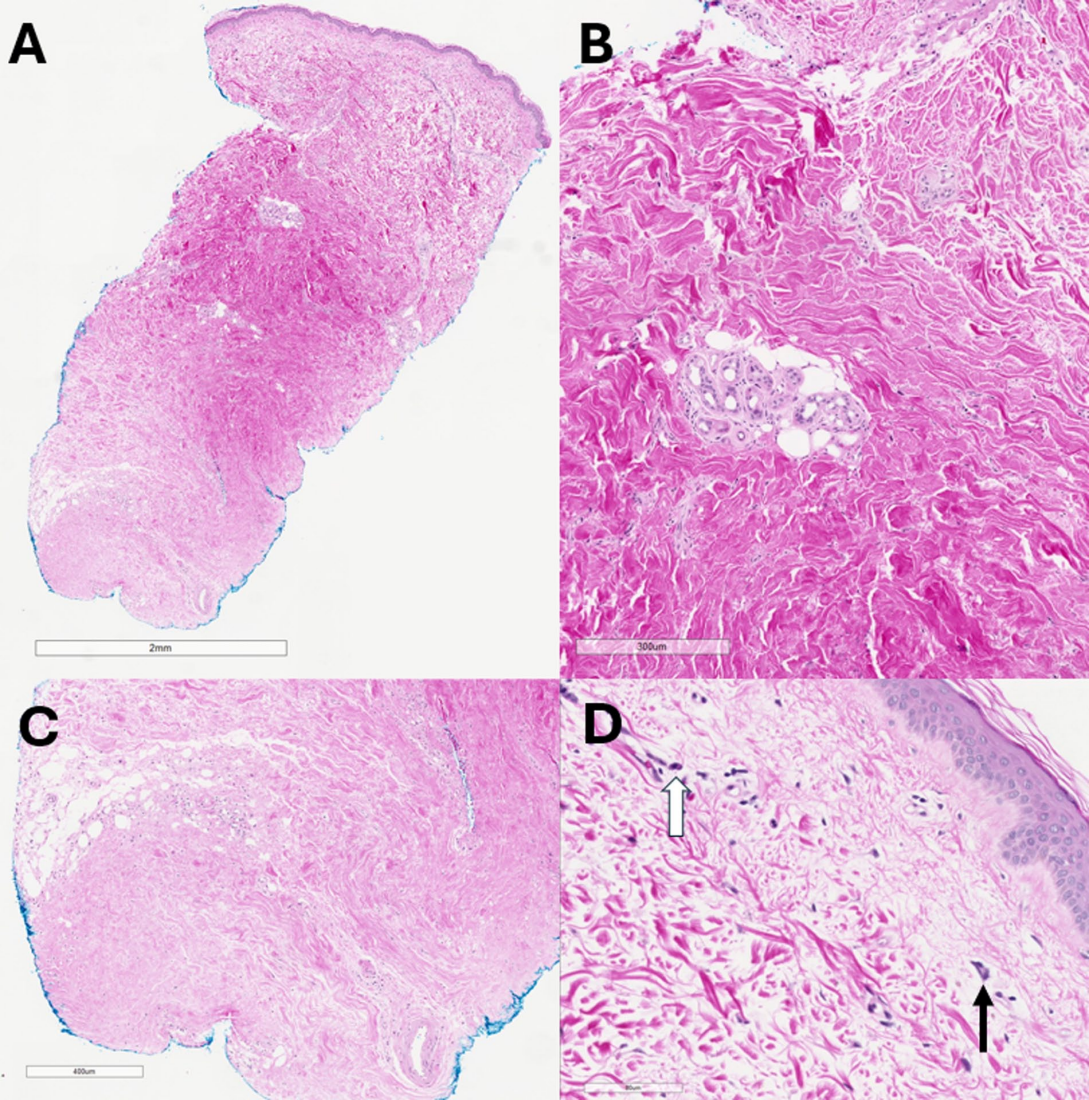
(a–d) Haematoxylin and eosin–stained punch biopsy showing (a) dense dermal sclerosis with mild superficial and deep lymphoplasmacytic inflammation. (b) Compressed eccrine coils with loss of peri-adnexal fat. (c) Loss of normal subcutaneous fat with focal fat necrosis. (d) Atypical radiation fibroblasts (black arrow) and plasma cells (white arrow).

Punch biopsies showed skin with sclerotic dermal collagen bundles (Figure 2(a)) and loss of peri-adnexal adipose tissue (Figure 2(b)). Sclerosis extended into the adipose tissue of the breast, associated with focal fat necrosis (Figure 2(c)). Rare atypical dermal fibroblasts (Figure 2(d)) were compatible with the clinical history of prior radiation exposure. Atypical hyperplasia, in situ, and invasive malignancy were not identified.

The patient’s clinical presentation, history, and histopathology supported a diagnosis of RIM. She received intralesional injections of triamcinolone acetonide 10 mg/ml into the thickened areas of the right breast and was started on oral prednisone 50 mg daily, as well as topical clobetasol 0.05% ointment twice daily. Prednisone was rapidly tapered and discontinued after 2 weeks due to neurologic complications. Despite this, there was a marked reduction in associated erythema and some softening of the sclerotic bands, which continued to improve with repeat intralesional corticosteroid injections to the affected areas of the right breast. In addition to physical therapy, which focussed on soft tissue mobilisation and fascial stretching, she also received a right serratus block by physiatry, providing noticeable pain relief to her right breast and chest wall. To date, her RIM is stable with no clinical evidence of active inflammation or necessitating further therapies.

## Discussion

RIM is a rare complication of radiotherapy, typically affecting the breast, characterised by inflammatory fibrosis of the dermis and/or subcutaneous tissues.^1^ It is estimated that 1 in 378 breast cancer patients develop RIM,^2^ which is considerably higher than the incidence of idiopathic morphoea in the non-irradiated population at 2.7:100,000 persons/year.^3^ An updated review of the literature revealed 120 cases of RIM involving the breast in females since 1989 (Supplemental Table S1). The average age of RIM onset was 61.1 years (range: 40–86 years) and most cases presented within a year of radiotherapy completion. Neither age, total radiation dose, nor fractionation of radiotherapy correlates with the incidence or severity of RIM.^4^

RIM can lead to substantial disfiguration and pain, greatly impacting the patient’s quality of life. Symptom onset usually manifests abruptly within a year after radiotherapy, although intervals ranging from 1 month to 32 years have been described.^1,2^ Typical clinical features include inflammatory erythematous and oedematous plaques accompanied by painful induration and shrinkage of the affected tissue.^1^ These changes are predominantly confined to radiation-exposed areas but may spread to other nearby tissues. Because presentation is non-specific, RIM is frequently misdiagnosed as cellulitis, chronic radiation dermatitis, or cancer recurrence.^1,4^ Therefore, a biopsy is essential for appropriate diagnosis and management. Inflammation is the predominant histological feature in early lesions, which show abundant perivascular and peri-adnexal infiltration of lymphocytes.^4^ By contrast, later lesions exhibit widespread fibrosis characterised by extensive thickened and sclerotized collagen fibres.

Response to RIM treatment is highly variable and challenging, as the natural history and pathophysiology of RIM are poorly understood. The general consensus is that a dysregulated immune-mediated response alters appropriate fibroproliferation and collagen synthesis in genetically susceptible individuals.^5^ Emerging research suggests that quiescent fibroblasts undergo aberrant phenotypic changes following radiation, triggering the inflammatory fibrosis seen in RIM.^6^ Therefore, management of RIM is similar to treating idiopathic morphoea, focussing on halting active disease and preventing progression.^7^ Because active disease responds more favourably to intervention, early recognition and treatment initiation are critical.^8^ Initial strategies include topical high-potency corticosteroids, topical calcineurin inhibitors, intralesional, and/or oral corticosteroids. In more resistant or extensive cases of RIM, phototherapy, systemic immunosuppressant agents such as methotrexate, and surgery may be employed.

Pain management is also crucial in RIM. As evident in the presented case, integrating approaches similar to those used for post-mastectomy pain, including regional nerve blocks like thoracic paravertebral or serratus anterior plane blocks, can provide substantial relief while addressing the underlying RIM. Evidence from the post-mastectomy pain literature supports the role of focal anaesthetic techniques in mitigating chronic neuropathic pain, suggesting a broader applicability in radiation-induced syndromes.^9,10^ Given the overlap in neuropathic mechanisms between post-mastectomy pain and RIM, these interventional strategies should be considered as part of a multimodal, multidisciplinary management plan for patients with RIM.

Our case highlights RIM as a painful and disfiguring radiation reaction commonly affecting the breast. Although uncommon, RIM should be considered as a possible complication of radiotherapy with equal suspicion to malignancy recurrence or radiation dermatitis, as early treatment and multidisciplinary care to manage pain can greatly improve a patient’s quality of life.

## Supplemental Material

sj-pdf-1-sco-10.1177_2050313X261463846 – Supplemental material for Radiation-induced morphoea in the setting of previous breast cancer: A case report Supplemental material, sj-pdf-1-sco-10.1177_2050313X261463846 for Radiation-induced morphoea in the setting of previous breast cancer: A case report by Sydney A. Yee, David M. Langelier, Kyla Taylor and Lynne H. Robertson in SAGE Open Medical Case Reports
