# Supplementary material for: Radiation-induced morphoea in the setting of previous breast cancer: A case report
Source: SAGE Open Med Case Rep. 2026 Jul 8;14:2050313X261463846. doi: 10.1177/2050313X261463846 (PMC13346743; doi:10.1177/2050313X261463846)
Supplement: sj-pdf-1-sco-10.1177_2050313X261463846 – Supplemental material for Radiation-induced morphoea in the setting of previous breast cancer: A case report [file sj-pdf-1-sco-10.1177_2050313X261463846.pdf]

SUPPLEMENTAL

TABLE S1. Reports of radiation-induce morphoea associated with breast cancer published since 1989. Updated and adapted from Gonzalez-Ericsson et al.[1], Partl et al.[2], and Joly-Chevrier et al.[3]. Included reports were limited to adults (≥18-years-old), females, and English text. RIM = radiation-induced morphoea, PUVA = psoralen ultraviolet-A

Author	Cases	Mean Age (Year)	Primary Carcinoma	Interval Between Radiotherapy and RIM	Skin Disease Beyond Irradiated Area	Treatment Modality	Treatment Efficacy
Clover et al.[4]	7	56.6	Invasive	1-10 years	2/7	No data	No data

Forbes et al.[5]	1	48	Invasive	<18 months	1/1	D-penicillamine (250mg daily)	Skin softening; sclerodactyly progression; development of Raynaud's and arthropathy
Robertson et al.[6]	2	58.5	Invasive	<6-11 months	1/2	Mastectomy (Case 1); no treatment (Case 2)	No recurrence (Case 1);

							progression including involvement of hands and oesophagus at 27 months (Case 2)
Trattner et al.[7]	1	57	Invasive	6 weeks	1/1	Procaine penicillin (1.2 x 10^6 U/d IM) x 10 days; amoxicillin (1.5g/day) and	No improvement

						cloxacillin (2g/day) x 10 days	
Winkelman n et al.[8]	4	68.3	Invasive	1-6 months	0/4	Corticosteroids; wet compress; dicloxacillin sodium or erythromycin (1-3 months)	Improvement, but visible inflammation remained
Davis et al.[9]	6	52	Invasive	<12 months	0/6	Topical; intralesional; systemic therapy	No improvement

Mayr et al.[10]	1	49	Invasive	<7 months	0/1	Hydroxychloroquine (400mg daily); prednisone (20mg daily); pentoxifylline (400mg three times a day)	Improvement to contour changes and fibrosis
Gollob et al.[11]	1	54	Invasive	<12 months	0/1	Topical steroids	No data
Bleasel et al.[12]	4	50.8	Invasive	1-3 months	0/4	Topical steroids under occlusion for 4-5 months	No improvement

Schaffer et al.[13]	2	45	Invasive	8-32 years	0/2	Topical steroids and doxycycline (case 1); no treatment (case 2)	Normal skin at 12 months (case 1); improvement at 9 months (case 2)
Ullén & Björkholm[14]	1	67	*In situ*	2 months	1/1	No data	No data
Ardern-Jones &	1	73	*In situ*	5 years	1/1	Methotrexate (2.5mg weekly)	No data

Black[15]							
Reddy et al.[16]	1	75	*In situ*	4 months	1/1	Prednisone (60mg with taper to 15mg daily)	Improved skin changes
Dancey & Waters[17]	1	61	Invasive	<12 months	0/1	No data	No data
Dubner et al.[18]	1	55	Invasive	2 years	0/1	Mastectomy refused by patient	No data
Scope et al.[19]	4	55.5	2 Invasive; 2 *in situ*	4 months – 8 years	4/4	No data	No data
Seale et	1	55	Invasive	6 months	0/1	No data	No data

al.[20]							
Walsh et al.[21]	5	57.6	Invasive	<4-12 years	1/5	Topical clobetasol (case 2); mastectomy (case 5); no data (case 1, 4); no treatment (case 3)	Softening of skin over 5 years (case 2); no data (case 1, 3-5)
Cheah et al.[22]	1	57	Invasive	9 months	0/1	Topical and oral steroids; oral PUVA	No improvement
Herrmann et al.[23]	1	86	Invasive	18 months	0/1	0.1% topical methylprednisolone	Improved erythema

						aceponate and heparin-containing cream for 4 weeks; 10 day penicillin infusion; oral penicillin G for 3 weeks	after 2 months
Mosterd et al.[24]	1	53	Invasive	7 years	0/1	Topical class III steroids; PUVA	Improvement, but relapse months later

De Giorgi et al.[25]	1	68	Invasive	14 years	0/1	Mastectomy for concurrent angiosarcoma	No recurrence
Afonso-Afonso et al.[26]	1	51	Invasive	11 months	0/1	Topical steroids	Improvement
Clark & Weschter[27]	2	71.5	Invasive	5-26 months	0/2	Topical corticosteroids (case 1); no treatment (case 2)	No data

Kushi & Csuka[28]	1	69	Invasive	7 months	1/1	Topical calcipotriol and betamethasone dipropionate; minocycline (100mg twice a day); methotrexate (7.5mg titrated to 12.5mg weekly)	Resolved inflammation; thickened skin unchanged
Froman et al.[29]	1	78	Invasive	2 years	0/1	Topical steroids	Improvement of erythema

							and skin thickening
Wernicke et al.[30]	1	66	Invasive	18 months	0/1	No treatment	Spontaneous skin softening
Laetsch et al.[31]	3	63.7	Invasive	5-18 months	0/3	Systemic and topical corticosteroids (case 1); no treatment (case 2); topical	Improvement (case 1, 3); no data (case 2)

						corticosteroids and methotrexate (case 3)	
Alhathlool et al.[32]	1	64	Invasive	3 years	0/1	Penicillin 10 Mega IV three times a day for 14 days; topical calcipotriol cream; UVA1 for 15 days	Mild skin softening over 1 month
Newland & Marshman [33]	1	43	Invasive	<1 month	0/1	Narrowband UVB thrice weekly;	Skin softening; improved

						acitretin (10mg daily)	tenderness and arm mobility at 2 months
Morganroth et al.[34]	1	51	Invasive	6 years	0/1	0.5% clobetasol ointment daily	Improved erythema and palpable fullness
Lim et al.[35]	1	40	Invasive	7 months	0/1	Topical steroids; oral prednisolone	Skin softening;

						(30mg daily); PUVA twice weekly; UVA1	pain reduction
Hellen et al.[36]	1	53	Invasive	2 years	0/1	Topical clobetasol propionate under occlusion	Resolved blistering
Yanaba et al.[37]	1	67	Invasive	3 months	1/1	Topical corticosteroids; tacrolimus; narrow-band UVB; hydroxychloroquine	No improvement

						sulphate (200mg) for 6 months	
Dyer et al.[38]	2	69	Invasive	3-4 months	0/2	No data	No data
Rafique et al.[39]	3	53.7	Invasive	3.5 months – 3 years	1/3	Mastectomy with reconstruction	No recurrence or progression
Fruchter et al.[40]	21	57.9	Invasive	2 months – 11 years	11/21	Various therapies including topical, intralesional, and systemic steroids,	Variable levels of improvement with greatest

						calcipotriene, methotrexate, and phototherapy	response to systemic therapies
Gonzalez-Ericsson et al.[1]	1	44	Invasive	15 months	0/1	Oral prednisone (40mg/ day); methotrexate (25mg/week); PUVA for 2 months	Improved pain and some skin softening
Franco et al.[41]	1	62	Invasive	3 months	1/1	Pentoxifylline (400mg) three times	Improvement in erythema

						a day; oral vitamin E (400IU) daily	and induration
Friedman et al.[42]	3	52.7	Invasive	4-7 years	0/3	IV methylprednisolone (500mg) for 3 days and topical Derma Gran B paste (case 1); methotrexate and UVA1 (case 2); UVA1 and topical tacrolimus 0.1%	No recurrence or progression

						daily for 3 months (case 3)	
Kim et al.[43]	1	53	Invasive	1 year	1/1	Oral prednisone (40mg with taper to 10mg daily over 3 months); tofacitinib (5mg three times a day); intermittent extracorporeal photopheresis twice weekly;	Improved skin induration and range of movement

						methotrexate (10mg weekly) for 3 months	
Papanikolaou et al.[44]	1	78	Invasive	4 months	0/1	Oral prednisolone; topical corticosteroids; doxycycline (100mg daily); methotrexate (15mg weekly); hydroxychloroquine (200mg two times a	Improvement in pain and healing of ulcers with photodynamic therapy only

						day); photodynamic therapy	
Partl et al.[45]	1	72	Invasive	3 months	0/1	Topical steroids	No improvement
Diago et al.[46]	6	64.2	Invasive	4-16 years	0/6	Topical corticosteroid (case 1, 3); topical corticosteroid and tacrolimus (case 2); topical corticosteroid, UVB,	No data

						methotrexate (case 4); no treatment (case 5-6)	
Machan et al.[47]	1	64	Invasive	4 years	1/1	Topical corticosteroids	Improvement with residual atrophy and depigmentati on
Partl et al.[2]	6	66.2	Invasive	4-10 months	2/6	Topical steroids (case 1); topical steroids, calcineurin	Improved inflammation

						inhibitors, and methotrexate (case 2); topical steroids and UVA (case 3); no treatment (case 4-6)	
Gambichler et al.[48]	1	66	Invasive	No data	0/1	IV prednisolone (1000mg) for 3 days every 4 weeks; subcutaneous	Improvement of skin fibrosis and resolution of erythema

						methotrexate (15mg) weekly	
Perna et al.[49]	1	60s	Invasive	17 years	0/1	No data	No data
Belzer et al.[50]	1	64	Invasive	6 months	1/1	Topical corticosteroids; methotrexate	Improvement
Zahn et al.[51]	1	68	Invasive	6 months	0/1	Topical corticosteroids	No improvement
Finnegan et al.[52]	3	65	Invasive	1-5 years	0/3	Topical mometasone	Skin softening

						furoate and calcipotriol (case 1,3); topical betamethasone/calcipotriol and clobetasol propionate (case 2)	
Titan et al.[53]	1	74	Invasive	7 months	0/1	Triamcinolone injections; topical agents; ultraviolet therapy;	Improved pain and range of motion post-

						mastectomy and reconstruction	reconstructio n
Panaitescu et al.[54]	1	77	Invasive	4 years	1/1	Prednisone; topical corticosteroids; topical vitamin D; phototherapy; oral mycophenolate mofetil (1g) two times a day	Improvement of pain and re-epithelialized ulcerated areas
Our Case	1	70	Invasive	18 months	0/1	Intralesional corticosteroid	Improved erythema

						injections (10mg/mL); topical clobetasol 0.05% ointment; prednisone (50mg) daily discontinued after 2 weeks due to side effects	and pain; some softening of skin

Supplemental Table References

1. Gonzalez-Ericsson PI, Estrada MV, Al-Rohil R, Sanders ME. Post-irradiation morphoea of the breast: a case report and review of the literature. *Histopathology*. Jan 2018;72(2):342-350. doi:10.1111/his.13343
2. Partl R, Regitnig P, Lukasiak K, Winkler P, Kapp KS. Incidence of Morphea following Adjuvant Irradiation of the Breast in 2,268 Patients. *Breast Care (Basel)*. Jun 2020;15(3):246-252. doi:10.1159/000502030
3. Joly-Chevrier M, Gelinas A, Ghazal S, et al. Morphea, Eosinophilic Fasciitis and Cancer: A Scoping Review. *Cancers (Basel)*. Sep 7 2023;15(18)doi:10.3390/cancers15184450
4. Colver GB, Rodger A, Mortimer PS, Savin JA, Neill SM, Hunter JA. Post-irradiation morphoea. *Br J Dermatol*. Jun 1989;120(6):831-5. doi:10.1111/j.1365-2133.1989.tb01382.x
5. Forbes AM, Woodrow JC, Verbov JL, Graham RM. Carcinoma of breast and scleroderma: four further cases and a literature review. *Br J Rheumatol*. Feb 1989;28(1):65-9. doi:10.1093/rheumatology/28.1.65

6. Robertson JM, Clarke DH, Pevzner MM, Matter RC. Breast conservation therapy. Severe breast fibrosis after radiation therapy in patients with collagen vascular disease. *Cancer*. Aug 1 1991;68(3):502-8. doi:10.1002/1097-0142(19910801)68:3<502::aid-cncr2820680310>3.0.co;2-v
7. Trattner A, Figer A, David M, Lurie H, Sandbank M. Circumscribed scleroderma induced by postlumpectomy radiation therapy. *Cancer*. Nov 15 1991;68(10):2131-3. doi:10.1002/1097-0142(19911115)68:10<2131::aid-cncr2820681007>3.0.co;2-y
8. Winkelmann RK, Grado GL, Quimby SR, Connolly SM. Pseudosclerodermatous panniculitis after irradiation: an unusual complication of megavoltage treatment of breast carcinoma. *Mayo Clin Proc*. Feb 1993;68(2):122-7. doi:10.1016/s0025-6196(12)60158-x
9. Davis DA, Cohen PR, McNeese MD, Duvic M. Localized scleroderma in breast cancer patients treated with supervoltage external beam radiation: radiation port scleroderma. *J Am Acad Dermatol*. Dec 1996;35(6):923-7. doi:10.1016/s0190-9622(96)90116-4

10. Mayr NA, Riggs CE, Saag KG, Wen BC, Pennington EC, Hussey DH. Mixed connective tissue disease and radiation toxicity. *Cancer*. 1997;79(3):612-618. doi:10.1002/(sici)1097-0142(19970201)79:3<612::Aid-cncr26>3.0.Co;2-5
11. Gollob MH, Dekoven JG, Bell MJ, Assaad D, Rao J. Postradiation morphea. *J Rheumatol*. Nov 1998;25(11):2267-9.
12. Bleasel NR, Stapleton KM, Commens C, Ahern VA. Radiation-induced localized scleroderma in breast cancer patients. *Australas J Dermatol*. May 1999;40(2):99-102. doi:10.1046/j.1440-0960.1999.00330.x
13. Schaffer JV, Carroll C, Dvoretsky I, Huether MJ, Girardi M. Postirradiation morphea of the breast presentation of two cases and review of the literature. *Dermatology*. 2000;200(1):67-71. doi:10.1159/000018322
14. UlIÉN H, BjÖRkholm E. Localized scleroderma in a woman irradiated at two sites for endometrial and breast carcinoma: A case history and a review of the literature. *International Journal of Gynecological Cancer*. 2003;13(1):77-82. doi:10.1136/ijgc-00009577-200301000-00014

15. Ardern-Jones MR, Black MM. Widespread morphoea following radiotherapy for carcinoma of the breast. *Clin Exp Dermatol*. Mar 2003;28(2):160-2. doi:10.1046/j.1365-2230.2003.01186.x

16. Reddy SM, Pui JC, Gold LI, Mitnick HJ. Postirradiation morphea and subcutaneous polyarteritis nodosa: case report and literature review. *Semin Arthritis Rheum*. Apr 2005;34(5):728-34. doi:10.1016/j.semarthrit.2004.11.004

17. Dancey AL, Waters RA. Morphea of the breast. Two case reports and discussion of the literature. *J Plast Reconstr Aesthet Surg*. 2006;59(10):1114-7. doi:10.1016/j.bjps.2006.01.018

18. Dubner S, Bovi J, White J, Susnik B. Postirradiation morphea in a breast cancer patient. *Breast J*. Mar-Apr 2006;12(2):173-6. doi:10.1111/j.1075-122X.2006.00229.x

19. Scope A, Sadetzki S, Sidi Y, et al. Breast cancer and scleroderma. *Skinmed*. Jan-Feb 2006;5(1):18-24. doi:10.1111/j.1540-9740.2006.04448.x

20. Seale M, Koh W, Henderson M, Drummond R, Cawson J. Imaging surveillance of the breast in a patient diagnosed with scleroderma after breast-conserving surgery and radiotherapy. *Breast J*. Jul-Aug 2008;14(4):379-81. doi:10.1111/j.1524-4741.2008.00603.x

21. Walsh N, Rheaume D, Barnes P, Tremaine R, Reardon M. Postirradiation morphea: an underrecognized complication of treatment for breast cancer. *Hum Pathol*. Nov 2008;39(11):1680-8. doi:10.1016/j.humpath.2008.04.010

22. Cheah NL, Wong DW, Chetiyawardana AD. Radiation-induced morphea of the breast: a case report. *J Med Case Rep*. Apr 30 2008;2:136. doi:10.1186/1752-1947-2-136

23. Herrmann T, Gunther C, Csere P. Localized morphea--a rare but significant secondary complication following breast cancer radiotherapy. Case report and review of the literature on radiation reaction among patients with scleroderma/morphea. *Strahlenther Onkol*. Sep 2009;185(9):603-7. doi:10.1007/s00066-009-2051-3

24. Mosterd K, Winnepenninckx V, Vermeulen A, van Neer PA, van Neer FJ, Frank J. Morphea following surgery and radiotherapy: an evolving problem. *J Eur Acad Dermatol Venereol*. Sep 2009;23(9):1099-101. doi:10.1111/j.1468-3083.2009.03097.x

25. de Giorgi V, Santi R, Grazzini M, et al. Synchronous angiosarcoma, melanoma and morphea of the breast skin 14 years after radiotherapy for mammary carcinoma. *Acta Derm Venereol*. May 2010;90(3):283-6. doi:10.2340/00015555-0841

26. Afonso-Afonso FJ, Arevalo MP, Cerecedo FC, De Paz Arias L, Tonder CD. Post-Irradiation Morphea in Breast Cancer: An Uncommon Differential Diagnosis to Keep in Mind. *World J Oncol*. Dec 2010;1(6):250-251. doi:10.4021/wjon264w

27. Clark CJ, Wechter D. Morphea of the breast--an uncommon cause of breast erythema. *Am J Surg*. Jul 2010;200(1):173-6. doi:10.1016/j.amjsurg.2009.06.024

28. Kushi J, Csuka ME. Generalized morphea after breast cancer radiation therapy. *Case Rep Rheumatol*. 2011;2011:951948. doi:10.1155/2011/951948
29. Froman J, Landercasper J, Ellis R, De Maiffe B, Theede L. Red breast as a presenting complaint at a breast center: an institutional review. *Surgery*. Jun 2011;149(6):813-9. doi:10.1016/j.surg.2010.12.013
30. Wernicke AG, Goltser Y, Trichter S, et al. Morphea as a consequence of accelerated partial breast irradiation. *Clin Breast Cancer*. Mar 2011;11(1):67-70. doi:10.3816/CBC.2011.n.012
31. Laetsch B, Hofer T, Lombriser N, Lautenschlager S. Irradiation-induced morphea: x-rays as triggers of autoimmunity. *Dermatology*. 2011;223(1):9-12. doi:10.1159/000330324
32. Alhathlool A, Hein R, Andres C, Ring J, Eberlein B. Post-Irradiation Morphea: Case report and review of the literature. *J Dermatol Case Rep*. Sep 28 2012;6(3):73-7. doi:10.3315/jdcr.2012.1106
33. Newland K, Marshman G. Success treatment of post-irradiation morphoea with acitretin and narrowband UVB. *Australas J Dermatol*. May 2012;53(2):136-8. doi:10.1111/j.1440-0960.2011.00864.x

34. Morganroth PA, Dehoratius D, Curry H, Elenitsas R. Postirradiation Morphea: A Case Report With a Review of the Literature and Summary of the Clinicopathologic Differential Diagnosis. *Am J Dermatopathol*. Oct 4 2013;doi:10.1097/DAD.0b013e3181cb3fdd
35. Lim D, Johnston S, Novakovic L, Fearfield L. Radiation-induced morphoea treated with UVA-1 phototherapy. *Clin Exp Dermatol*. Jul 2014;39(5):612-5. doi:10.1111/ced.12345
36. Hellen R, Kiely C, Murad A, et al. Two cases of dermatoses koebnerizing within fields of previous radiotherapy. *Clin Exp Dermatol*. Dec 2014;39(8):900-3. doi:10.1111/ced.12421
37. Yanaba K, Umezawa Y, Nakagawa H. A case of radiation-induced generalized morphea with prominent mucin deposition and tenderness. *Am J Case Rep*. May 10 2015;16:279-82. doi:10.12659/AJCR.893481
38. Dyer BA, Hodges MG, Mayadev JS. Radiation-Induced Morphea: An Under-Recognized Complication of Breast Irradiation. *Clin Breast Cancer*. Aug 2016;16(4):e141-3. doi:10.1016/j.clbc.2016.05.001

39. Rafique B, McInerney N, Fitzgerald G, O'Hanlon D, Gilmore J, Kelly EJ. Post-irradiation morphea of the breast: does this pose an issue for reconstruction? *European Journal of Plastic Surgery*. 2016;40(1):67-70. doi:10.1007/s00238-016-1226-2
40. Fruchter R, Kurtzman DJB, Mazori DR, et al. Characteristics and treatment of postirradiation morphea: A retrospective multicenter analysis. *J Am Acad Dermatol*. Jan 2017;76(1):19-21. doi:10.1016/j.jaad.2016.08.059
41. Franco L, Hausauer AK, Patel RR, Guth AA, McLellan BN. Postirradiation morphea: unique presentation on the breast. *Cutis*. Nov 2018;102(5):E10-E12.
42. Friedman O, Barnea Y, Hafner A. Underdiagnosed and disfiguring - Radiation-induced morphea following breast cancer treatment. *Breast*. Jun 2018;39:97-100. doi:10.1016/j.breast.2018.04.006

43. Kim SR, Charos A, Damsky W, Heald P, Girardi M, King BA. Treatment of generalized deep morphea and eosinophilic fasciitis with the Janus kinase inhibitor tofacitinib. *JAAD Case Rep*. Jun 2018;4(5):443-445. doi:10.1016/j.jdcr.2017.12.003
44. Papanikolaou M, Tsianou Z, Skellett AM, Murphy J, Millington GWM. Radiotherapy-induced morphoea of the breast responding to photodynamic therapy. *Clin Exp Dermatol*. Jun 2018;43(4):506-508. doi:10.1111/ced.13420
45. Partl R, Regitnig P, Tauber G, Potscher M, Bjelic-Radisic V, Kapp KS. Radiation-induced morphea-a rare but severe late effect of adjuvant breast irradiation : Case report and review of the literature. *Strahlenther Onkol*. Nov 2018;194(11):1060-1065. Strahleninduzierte Morphea - eine seltene, aber schwere spate Folge der adjuvanten Brustbestrahlung : Fallbericht und Literaturubersicht. doi:10.1007/s00066-018-1336-9

46. Diago A, Llombart B, Requena C, Sanmartín O, Guillén C. Postirradiation Morphea in Patients With Breast Cancer: Possible Association With Other Autoimmune Diseases. *Actas Dermo-Sifiliográficas (English Edition)*. 2019;110(2):153-159. doi:10.1016/j.adengl.2019.01.006
47. Machan A, Oumakhir S, Khalidi M, Hjira N, Boui M. Radiation-induced morphea: autoimmunity as a risk factor. *Neth J Med*. Jan 2019;77(1):29-31.
48. Gambichler T, Scheel CH, Boms S. Radiation-induced morphea - a rare, but not to be dismissed, adverse effect of radiotherapy. *Dermatol Ther*. Sep 2021;34(5):e15041. doi:10.1111/dth.15041
49. Perna D, Margheim A, Schadt CR. Radiation-induced morphea and dystrophic calcinosis cutis of the breast. *Int J Dermatol*. Sep 2021;60(9):e373-e375. doi:10.1111/ijd.15483
50. Belzer A, McNiff JM, Leventhal JS. Skin eruption involving bilateral breasts following radiation therapy for invasive ductal carcinoma of the left breast. *Int J Womens Dermatol*. Jun 2022;8(2):e016. doi:10.1097/JW9.0000000000000016

51. Zahn CA, Feldmeyer L, Blum R, Mainetti C. Post-Irradiation Morphea of the Breast in a Patient with Subacute Cutaneous Lupus Erythematosus: Case Report and a Literature Review. *Case Rep Dermatol*. May-Aug 2022;14(2):144-150. doi:10.1159/000524514

52. Finnegan P, Kiely L, Gallagher C, et al. Radiation-induced morphea of the breast-A case series. *Skin Health Dis*. Feb 2023;3(1):e148. doi:10.1002/ski2.148

53. Titan A, Mohan AT, Tokuyama M, Mirbegian J, Bean GR, Lee GK. Radiation-Induced Morphea of the Breast Treated With Wide Local Excision and Abdominal Free Flap Breast Reconstruction. *Eplasty*. 2023;23:e50.

54. Panaitescu A, Nguyen H, Masson-Cote L, Fernandes CL. Extensive Morphea Following Adjuvant Radiotherapy for Breast Carcinoma-Case Report. *Curr Oncol*. Jan 18 2025;32(1)doi:10.3390/curroncol32010050
